# Supplementary material for: Dual-view jointly learning improves personalized drug synergy prediction
Source: Bioinformatics. 2024 Oct 18;40(10):btae604. doi: 10.1093/bioinformatics/btae604 (PMC11524890; doi:10.1093/bioinformatics/btae604)
Supplement: btae604_Supplementary_Data [file btae604_supplementary_data.pdf]

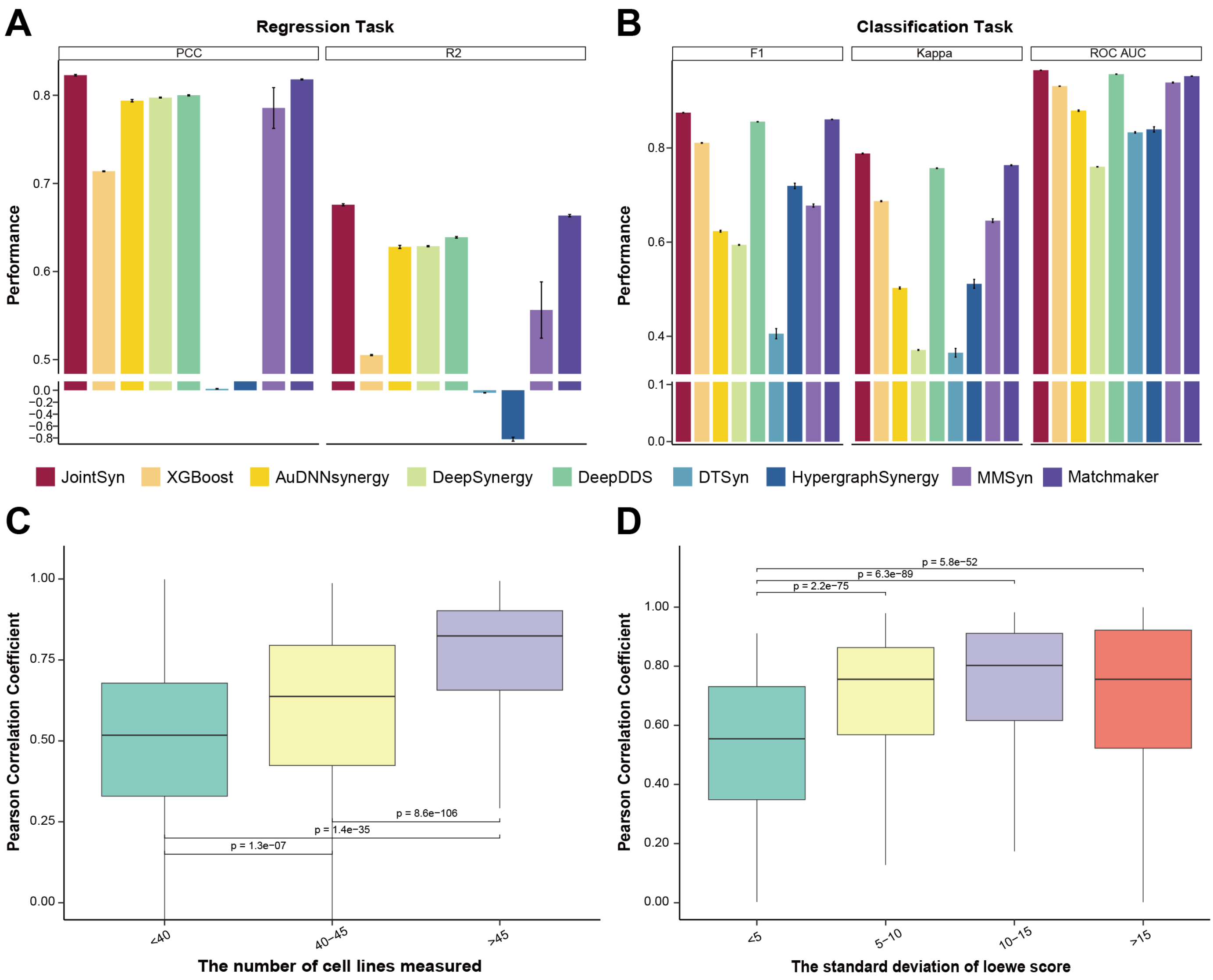

Supplementary Fig. 1 Evaluation of JointSyn on the NCI-ALMANAC benchmark dataset. A-B, Performance comparison for the regression and classification task on the NCI-ALMANAC benchmark dataset. Five-fold cross-validations were replicated 10 times to calculate the standard deviations (Error bars). **C-D**, Taken PCC of NCI-ALMANAC regression model as an example to discuss factors associated with the performance of each drug combination. **C**, The relationship between the PCC and the number of cell lines measured of each drug combination. **D**, The relationship between the PCC and the standard deviation of the real synergy score.

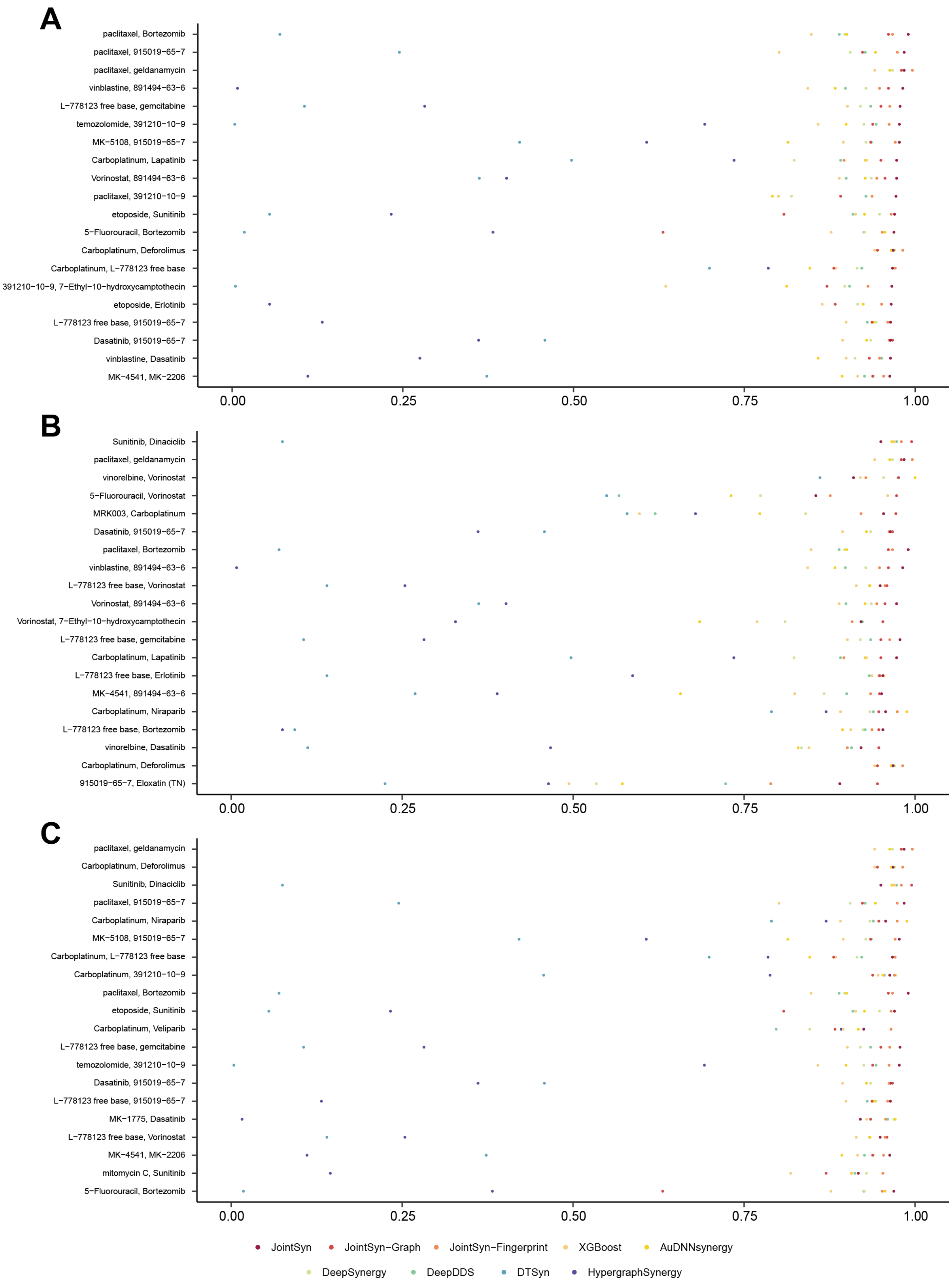

Supplementary Fig. 2 The contribution of two views by comparing them on each drug combination. A-C, The top 20 drug combinations by the JointSyn, JointSyn- Graph and JointSyn-Fingerprint respectively.

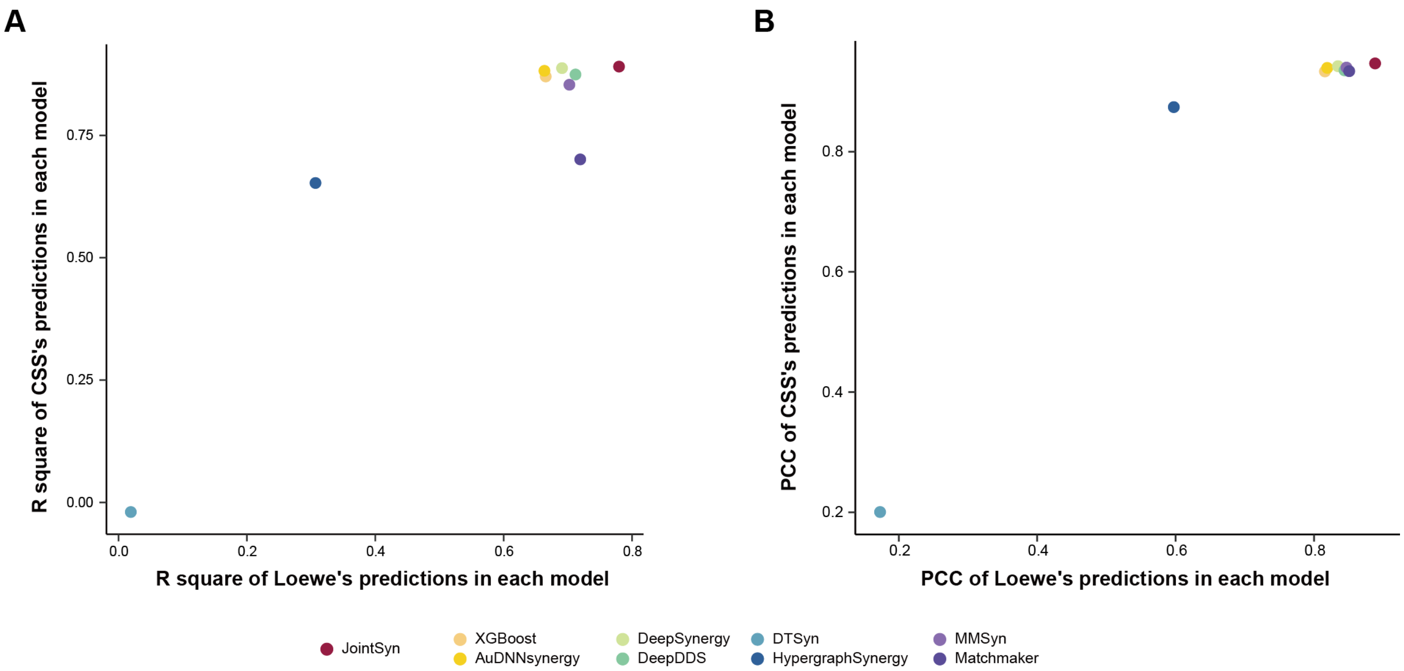

Supplementary Fig. 3 Model performance for predicting LOEWE synergy scores and CSS values. A, R2 of Loewe's predictions and CSS's predictions in each model. **B**, PCC of Loewe's predictions and CSS's predictions in each model.

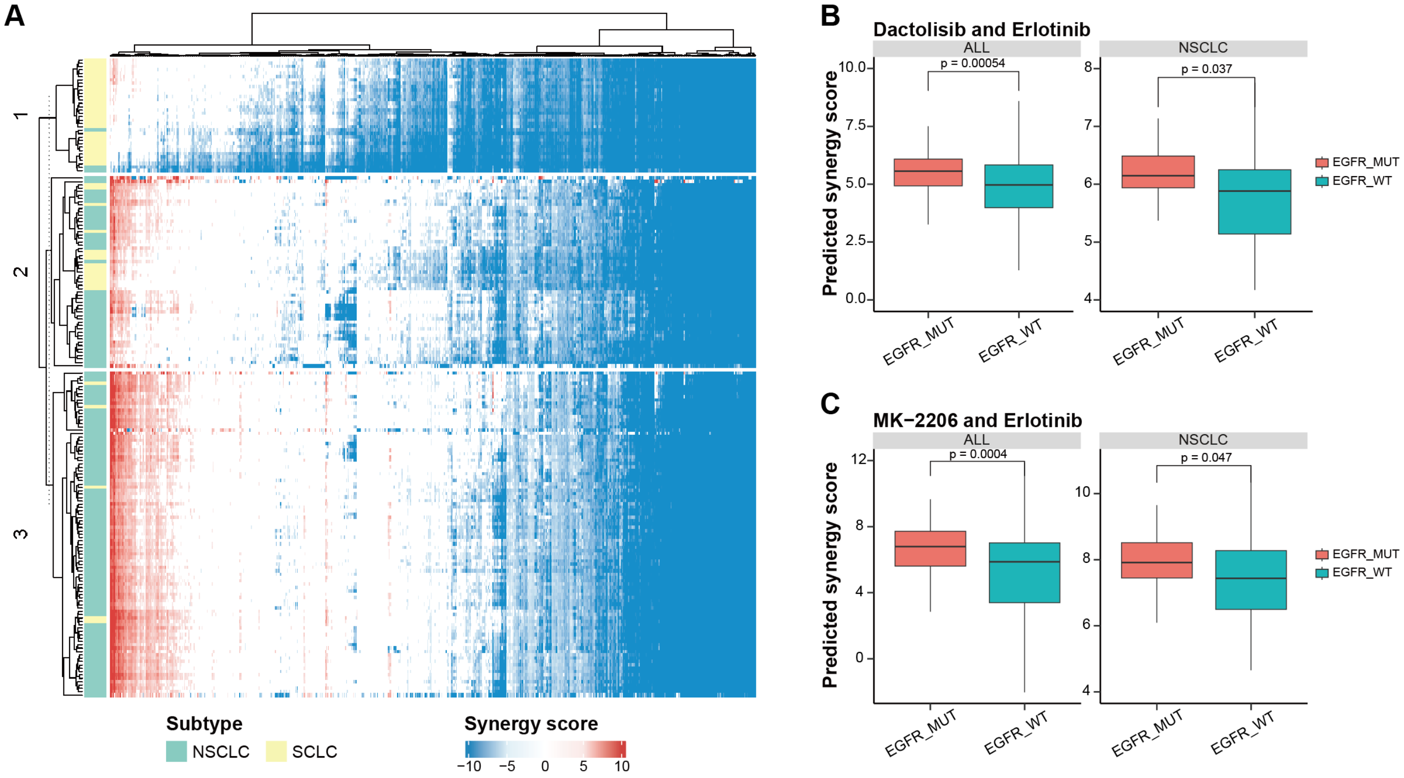

Supplementary Fig. 4 The predicted synergy scores of pan-cancers. A, The predicted synergy scores of all drug combinations on lung cell lines. **B**, The predicted synergy scores in with and without EGFR mutations cell lines for all tumor or NSCLC on erlotinib and dactolisib. **C**, The predicted synergy scores in with and without EGFR mutations cell lines for all tumor or NSCLC on erlotinib and MK-2206.

Supplementary Table 1. The details of the drug synergy dataset.

	NCI-ALMANAC	O’neil
Number of samples in the raw dataset	296166	82712
Number of samples after preprocessing	236190	12033
Number of drugs	103	38
Number of cell lines	46	34
Number of drug combinations	5317	583

Supplementary Table 2. The detailed parameter settings of JointSyn.

Hyperparameters	Values
Loss function of the classification task	Cross entropy
Loss function of the regression task	MSE
Activation function of output layer in the classification task	Softmax
Activation function of output layer in the regression task	Linear
Activation function of hidden layers	Relu
Maximum training epoch	1000
Batch size	**128,** 256, 512
Learning rate	**0.0001**, 0.0003, 0.0005, 0.001
Dropout rate	0,**0.2**,0.5
Graph Net hidden size	[256,256,256], [256,256,512], [512,512,512], **[512,512,1024]**, [1024,1024, 1024]
Global Pooling method	**max nodes**, mean nodes
Number of attention heads	[5,5,1], [5,5,2], **[10,5,1]**, [10,5,2]
Fingerprint Net hidden size	[1024,512], [1024,1024], **[2048,1024]**, [2048,2048]
**Prediction net hidden size**	[3072,512,256,128], [3072,1024,256,128], **[3072,1024,512,128]**, [3072,1024,512,256]

Supplementary Table 3. The performance of the four drug joint methods by cross-validation.

	R2	PCC	Kappa	F1	MSE	RMSE	ROC AUC	PR AUC	BACC	Precision	Recall
Super Edge	**0.705**	**0.851**	**0.8**	**0.865**	**102.586**	**10.128**	**0.964**	**0.933**	**0.9**	**0.865**	**0.865**
	**(0.701,0.708)**	**(0.848,0.853)**	**(0.797,0.803)**	**(0.863,0.867)**	**(101.333,103.838)**	**(10.067,10.19)**	**(0.963,0.964)**	**(0.932,0.934)**	**(0.898,0.902)**	**(0.862,0.868)**	**(0.86,0.87)**
All Node	0.46	0.687	0.624	0.747	187.656	13.699	0.903	0.821	0.813	0.743	0.751
	(0.456,0.464)	(0.685,0.69)	(0.617,0.631)	(0.743,0.752)	(186.375,188.937)	(13.652,13.745)	(0.899,0.907)	(0.816,0.827)	(0.809,0.816)	(0.738,0.748)	(0.745,0.758)
One Super Node	0.694	0.843	0.795	0.862	106.172	10.304	0.962	0.931	0.898	0.861	0.862
	(0.691,0.698)	(0.842,0.845)	(0.791,0.799)	(0.859,0.865)	(104.939,107.405)	(10.244,10.363)	(0.961,0.963)	(0.929,0.933)	(0.895,0.9)	(0.857,0.866)	(0.858,0.867)
Three Super Nodes	0.695	0.844	0.795	0.862	105.886	10.29	0.962	0.931	0.897	0.863	0.86
	(0.693,0.698)	(0.843,0.845)	(0.791,0.799)	(0.858,0.865)	(105.049,106.722)	(10.249,10.331)	(0.96,0.963)	(0.929,0.933)	(0.894,0.9)	(0.86,0.866)	(0.853,0.867)

There are mean values and 95% confidence intervals of ten replicates. The best performance is shown in bold.

Supplementary Table 4. The comparison of performance of JointSyn and other methods on the O'NEIL dataset by cross-validation.

	R2	PCC	F1	Kappa	ROC AUC	MSE	RMSE	PR AUC	BACC	Precision	Recall
JointSyn	**0.78**	**0.888**	**0.877**	**0.819**	**0.968**	**76.562**	**8.75**	**0.94**	**0.909**	**0.88**	**0.876**
	**(0.777,0.782)**	**(0.886,0.89)**	**(0.875,0.88)**	**(0.814,0.823)**	**(0.967,0.969)**	**(75.612,77.511)**	**(8.695,8.804)**	**(0.938,0.942)**	**(0.907,0.911)**	**(0.874,0.885)**	**(0.872,0.88)**
XGBoost	0.666	0.816	0.852	0.782	0.959	116.264	10.782	0.926	0.887	0.868	0.836
	(0.663,0.668)	(0.814,0.817)	(0.85,0.854)	(0.78,0.785)	(0.959,0.96)	(115.325,117.204)	(10.739,10.826)	(0.924,0.927)	(0.886,0.889)	(0.866,0.871)	(0.833,0.839)
AudnnSynergy	0.664	0.819	0.688	0.574	0.891	119.528	10.933	0.805	0.768	0.795	0.607
	(0.66,0.667)	(0.817,0.821)	(0.685,0.691)	(0.57,0.577)	(0.89,0.892)	(118.188,120.868)	(10.871,10.994)	(0.804,0.806)	(0.766,0.77)	(0.792,0.798)	(0.602,0.612)
DeepSynergy	0.691	0.835	0.714	0.597	0.892	107.43	10.365	0.815	0.784	0.798	0.646
	(0.687,0.694)	(0.832,0.837)	(0.712,0.717)	(0.593,0.6)	(0.892,0.893)	(106.182,108.678)	(10.304,10.425)	(0.814,0.815)	(0.782,0.786)	(0.796,0.8)	(0.642,0.65)
DeepDDS	0.712	0.844	0.853	0.782	0.958	100.098	10.005	0.921	0.89	0.855	0.85
	(0.709,0.715)	(0.842,0.846)	(0.85,0.855)	(0.778,0.787)	(0.958,0.959)	(99.026,101.17)	(9.951,10.058)	(0.919,0.923)	(0.888,0.892)	(0.85,0.86)	(0.846,0.854)
DTSyn	0.019	0.173	0.803	0.705	0.928	340.859	18.462	0.868	0.855	0.79	0.815
	(0.017,0.021)	(0.162,0.184)	(0.8,0.805)	(0.701,0.709)	(0.927,0.929)	(340.213,341.504)	(18.445,18.48)	(0.867,0.87)	(0.853,0.858)	(0.783,0.797)	(0.806,0.825)
HypergraphSynergy	0.307	0.597	0.814	0.72	0.927	240.859	15.519	0.872	0.866	0.787	0.843
	(0.301,0.313)	(0.591,0.604)	(0.81,0.818)	(0.713,0.727)	(0.923,0.93)	(238.644,243.074)	(15.448,15.59)	(0.866,0.878)	(0.863,0.87)	(0.778,0.796)	(0.836,0.851)
MMSyn	0.702	0.847	0.851	0.786	0.958	103.52	10.174	0.92	0.891	0.859	0.843
	(0.698,0.707)	(0.844,0.849)	(0.849,0.853)	(0.782,0.79)	(0.956,0.96)	(101.963,105.077)	(10.097,10.25)	(0.916,0.925)	(0.889,0.892)	(0.853,0.865)	(0.839,0.847)
Matchmakerr	0.719	0.851	0.837(0.833,0.841)	0.758	0.948	97.568	9.877	0.905	0.879	0.837	0.836
	(0.715,0.723)	(0.849,0.853)	(0.833,0.841)	(0.752,0.764)	(0.946,0.949)	(96.194,98.942)	(9.808,9.947)	(0.902,0.908)	(0.876,0.882)	(0.832,0.842)	(0.831,0.842)

There are mean values and 95% confidence intervals of ten replicates. The best performance is shown in bold.

Supplementary Table 5. The comparison of performance of JointSyn and other methods on the NCI-ALMANAC dataset by cross-validation.

	R2	PCC	F1	Kappa	ROC AUC	MSE	RMSE	PR AUC	BACC	Precision	Recall
JointSyn	**0.676**	**0.823**	**0.875**	**0.788**	**0.955**	**67.548**	**8.219**	**0.941**	**0.894**	**0.877**	**0.873**
	**(0.675,0.677)**	**(0.822,0.824)**	**(0.875,0.876)**	**(0.787,0.789)**	**(0.955,0.955)**	**(67.303,67.794)**	**(8.204,8.234)**	**(0.94,0.941)**	**(0.893,0.894)**	**(0.876,0.878)**	**(0.872,0.874)**
XGBoost	0.505	0.714	0.811	0.687	0.922	103.076	10.153	0.894	0.84	0.84	0.784
	(0.504,0.506)	(0.713,0.714)	(0.81,0.812)	(0.686,0.688)	(0.921,0.922)	(102.94,103.211)	(10.146,10.159)	(0.894,0.895)	(0.839,0.84)	(0.839,0.841)	(0.783,0.785)
AudnnSynergy	0.628	0.794	0.623	0.503	0.87	132.151	11.496	0.767	0.727	0.796	0.512
	(0.626,0.63)	(0.793,0.795)	(0.621,0.625)	(0.5,0.505)	(0.869,0.872)	(131.52,132.782)	(11.468,11.523)	(0.765,0.768)	(0.725,0.728)	(0.792,0.801)	(0.509,0.515)
DeepSynergy	0.629	0.797	0.594	0.371	0.753	77.325	8.793	0.698	0.679	0.688	0.523
	(0.628,0.629)	(0.797,0.798)	(0.594,0.595)	(0.37,0.372)	(0.753,0.754)	(77.18,77.47)	(8.785,8.802)	(0.697,0.698)	(0.678,0.679)	(0.687,0.689)	(0.522,0.525)
DeepDDS	0.639	0.8	0.856	0.757	0.947	75.256	8.675	0.929	0.877	0.864	0.849
	(0.638,0.64)	(0.799,0.801)	(0.855,0.856)	(0.756,0.758)	(0.946,0.947)	(75.049,75.462)	(8.663,8.687)	(0.928,0.929)	(0.877,0.878)	(0.862,0.865)	(0.847,0.85)
DTSyn	-0.044	0.023	0.406	0.365	0.825	284.804	16.876	0.455	0.641	0.619	0.303
	(-0.046,-0.041)	(0.017,0.028)	(0.395,0.416)	(0.356,0.374)	(0.824,0.826)	(284.033,285.576)	(16.853,16.899)	(0.448,0.463)	(0.634,0.648)	(0.599,0.639)	(0.286,0.321)
HypergraphSynergy	-0.82	0.398	0.72	0.511	0.831	378.935	19.465	0.717	0.758	0.695	0.746
	(-0.854,-0.785)	(0.395,0.401)	(0.714,0.725)	(0.502,0.521)	(0.826,0.837)	(371.843,386.028)	(19.283,19.647)	(0.708,0.726)	(0.754,0.763)	(0.688,0.701)	(0.737,0.755)
MMSyn	0.556	0.786	0.678	0.645	0.929	91.08	9.533	0.738	0.802	0.734	0.63
	(0.524,0.588)	(0.762,0.809)	(0.674,0.681)	(0.642,0.649)	(0.928,0.93)	(84.426,97.734)	(9.196,9.87)	(0.735,0.741)	(0.801,0.804)	(0.728,0.74)	(0.626,0.633)
Matchmakerr	0.663	0.818	0.861	0.763	0.943	70.135	8.375	0.926	0.882	0.86	0.862
	(0.662,0.665)	(0.817,0.819)	(0.86,0.861)	(0.762,0.764)	(0.942,0.943)	(69.845,70.426)	(8.357,8.392)	(0.925,0.926)	(0.881,0.882)	(0.858,0.861)	(0.86,0.863)

There are mean values and 95% confidence intervals of ten replicates. The best performance is shown in bold.

Supplementary Table 6. The comparison of performance of JointSyn and other methods on the O'NEIL dataset for unseen dataa to predict loewe synergy values.

	Random				PairOut				CellOut				DrugOut			
	R2	PCC	F1	Kappa	R2	PCC	F1	Kappa	R2	PCC	F1	Kappa	R2	PCC	F1	Kappa
JointSyn	**0.78**	**0.888**	**0.877**	**0.819**	**0.719**	**0.855**	**0.841**	**0.764**	0.413	**0.671**	**0.751**	**0.628**	-0.032	**0.194**	0.456	**0.141**
	**(0.777,0.782)**	**(0.886,0.89)**	**(0.875,0.88)**	**(0.814,0.823)**	**(0.713,0.725)**	**(0.851,0.859)**	**(0.836,0.845)**	**(0.757,0.771)**	(0.407,0.419)	**(0.667,0.675)**	**(0.747,0.756)**	**(0.62,0.636)**	(-0.055,-0.009)	**(0.162,0.227)**	(0.435,0.477)	**(0.108,0.174)**
XGBoost	0.666	0.816	0.852	0.782	0.503	0.711	0.789	0.692	0.39	0.642	0.721	0.589	-0.234	0.027	0.324	0.105
	(0.663,0.668)	(0.814,0.817)	(0.85,0.854)	(0.78,0.785)	(0.49,0.516)	(0.702,0.72)	(0.784,0.794)	(0.684,0.7)	(0.385,0.395)	(0.638,0.647)	(0.716,0.726)	(0.583,0.596)	(-0.273,-0.196)	(-0.022,0.076)	(0.303,0.344)	(0.074,0.135)
AudnnSynergy	0.664	0.819	0.688	0.574	0.538	0.739	0.645	0.521	0.392	0.644	0.602	0.455	-0.049	0.075	0.256	0.128
	(0.66,0.667)	(0.817,0.821)	(0.685,0.691)	(0.57,0.577)	(0.527,0.549)	(0.732,0.746)	(0.638,0.652)	(0.513,0.528)	(0.385,0.4)	(0.638,0.65)	(0.596,0.608)	(0.448,0.462)	(-0.075,-0.023)	(0.03,0.12)	(0.234,0.277)	(0.099,0.157)
DeepSynergy	0.691	0.835	0.714	0.597	0.558	0.757	0.679	0.55	0.103	0.571	0.634	0.451	**-0.031**	0.118	0.282	0.034
	(0.687,0.694)	(0.832,0.837)	(0.712,0.717)	(0.593,0.6)	(0.548,0.567)	(0.751,0.763)	(0.673,0.685)	(0.543,0.557)	(0.04,0.167)	(0.561,0.582)	(0.627,0.64)	(0.441,0.461)	**(-0.064,0.001)**	(0.065,0.17)	(0.247,0.317)	(0.011,0.057)
DeepDDS	0.712	0.844	0.853	0.782	0.612	0.784	0.797	0.701	0.398	0.656	0.743	0.622	-0.225	0.047	0.378	0.063
	(0.709,0.715)	(0.842,0.846)	(0.85,0.855)	(0.778,0.787)	(0.605,0.619)	(0.78,0.788)	(0.792,0.803)	(0.693,0.709)	(0.388,0.409)	(0.648,0.664)	(0.737,0.75)	(0.613,0.63)	(-0.323,-0.127)	(-0.04,0.134)	(0.346,0.41)	(0.037,0.089)
DTSyn	0.019	0.173	0.803	0.705	0.136	0.452	0.184	0.016	0.14	0.437	0.081	0.03	-0.126	-0.023	0.333	0.103
	(0.017,0.021)	(0.162,0.184)	(0.8,0.805)	(0.701,0.709)	(0.119,0.153)	(0.447,0.458)	(0.131,0.238)	(0.011,0.021)	(0.131,0.15)	(0.43,0.444)	(0.039,0.123)	(0.009,0.05)	(-0.17,-0.081)	(-0.08,0.035)	(0.273,0.393)	(0.069,0.138)
HypergraphSynergy	0.307	0.597	0.814	0.72	0.263	0.541	0.753	0.62	0.242	0.567	0.738	0.592	-0.301	0.075	**0.48**	0.071
	(0.301,0.313)	(0.591,0.604)	(0.81,0.818)	(0.713,0.727)	(0.25,0.277)	(0.525,0.558)	(0.746,0.76)	(0.608,0.633)	(0.234,0.251)	(0.558,0.575)	(0.733,0.742)	(0.584,0.6)	(-0.358,-0.244)	(-0.004,0.154)	**(0.444,0.516)**	(0.027,0.116)
MMSyn	0.702	0.847	0.851	0.786	0.637	0.8	0.787	0.694	0.281	0.578	0.727	0.606	-0.124	0.112	0.365	0.127
	(0.698,0.707)	(0.844,0.849)	(0.849,0.853)	(0.782,0.79)	(0.63,0.645)	(0.795,0.805)	(0.782,0.792)	(0.687,0.702)	(0.256,0.306)	(0.567,0.589)	(0.722,0.732)	(0.598,0.613)	(-0.184,-0.065)	(0.055,0.169)	(0.335,0.396)	(0.085,0.17)
Matchmakerr	0.719	0.851	0.837	0.758	0.625	0.795	0.765	0.65	**0.428**	0.666	0.746	0.62	-0.147	0.071	0.369	0.065
	(0.715,0.723)	(0.849,0.853)	(0.833,0.841)	(0.752,0.764)	(0.615,0.634)	(0.79,0.801)	(0.76,0.771)	(0.643,0.658)	**(0.42,0.436)**	(0.66,0.672)	(0.743,0.749)	(0.615,0.626)	(-0.181,-0.113)	(0.018,0.124)	(0.322,0.416)	(0.023,0.108)

There are mean values and 95% confidence intervals of ten replicates. The best performance is shown in bold.

Supplementary Table 7. The comparison of performance of JointSyn and other methods on the O'NEIL dataset for unseen data to predict CSS values.

	Random			PairOut			CellOut			DrugOut		
	RMSE	R2	PCC	RMSE	R2	PCC	RMSE	R2	PCC	RMSE	R2	PCC
JointSyn	**6.53**	**0.891**	**0.94**	**7.478**	**0.856**	**0.927**	15.865	0.353	0.607	19.647	-0.009	0.329
	**(6.468,6.591)**	**(0.889,0.893)**	**7(0.946,0.947)**	**(7.419,7.537)**	**(0.854,0.859)**	**(0.926,0.928)**	(15.588,16.142)	(0.33,0.375)	(0.59,0.623)	(19.341,19.953)	(-0.05,0.032)	(0.296,0.362)
XGBoost	7.104	0.87	0.934	8.857	0.798	0.894	**15.588**	0.375	0.628	20.908	-0.143	0.255
	(7.088,7.12)	(0.87,0.871)	(0.933,0.934)	(8.761,8.953)	(0.794,0.803)	(0.891,0.897)	**(15.277,15.9)**	(0.35,0.4)	(0.611,0.644)	(20.391,21.425)	(-0.201,-0.085)	(0.222,0.289)
AudnnSynergy	6.87	0.882	0.939	8.433	0.822	0.908	15.76	**0.378**	**0.643**	19.503	0.027	0.287
	(6.842,6.898)	(0.881,0.883)	(0.939,0.94)	(8.347,8.52)	(0.818,0.826)	(0.906,0.91)	(15.585,15.934)	**(0.364,0.392)**	**(0.633,0.654)**	(18.97,20.036)	(-0.011,0.066)	(0.246,0.328)
DeepSynergy	6.624	0.887	0.942	8.151	0.829	0.912	16.654	0.287	0.552	**18.767**	**0.08**	**0.359**
	(6.611,6.637)	(0.887,0.888)	(0.942,0.943)	(8.068,8.234)	(0.826,0.833)	(0.91,0.913)	(16.324,16.984)	(0.258,0.315)	(0.532,0.571)	**(18.45,19.084)**	**(0.055,0.105)**	**(0.331,0.388)**
DeepDDS	6.995	0.874	0.935	8.466	0.816	0.904	16.104	0.333	0.634	21.361	-0.199	0.275
	(6.965,7.025)	(0.873,0.876)	(0.935,0.936)	(8.401,8.53)	(0.813,0.818)	(0.903,0.905)	(15.842,16.366)	(0.312,0.355)	(0.617,0.65)	(20.238,22.484)	(-0.332,-0.066)	(0.217,0.332)
DTSyn	19.92	-0.02	0.2	17.291	0.232	0.55	17.464	0.216	0.522	21.867	-0.241	0.127
	(19.86,19.98)	(-0.026,-0.014)	(0.182,0.219)	(17.179,17.403)	(0.222,0.242)	(0.545,0.555)	(17.386,17.542)	(0.209,0.223)	(0.517,0.528)	(21.173,22.56)	(-0.324,-0.158)	(0.076,0.177)
HypergraphSynergy	11.614	0.653	0.874	11.394	0.666	0.845	15.967	0.345	0.602	54.123	-6.576	0.271
	(11.223,12.005)	(0.63,0.675)	(0.869,0.879)	(11.122,11.665)	(0.65,0.682)	(0.842,0.848)	(15.8,16.135)	(0.331,0.359)	(0.594,0.61)	(53.475,54.771)	(-6.727,-6.425)	(0.207,0.334)
MMSyn	7.502	0.854	0.94	9.263	0.777	0.9	21.357	-0.199	0.558	35.265	-2.341	0.071
	(7.466,7.537)	(0.852,0.855)	(0.939,0.94)	(9.097,9.429)	(0.769,0.784)	(0.898,0.903)	(20.151,22.562)	(-0.348,-0.051)	(0.54,0.577)	(31.395,39.135)	(-3.041,-1.64)	(-0.02,0.162)
Matchmakerr	10.786	0.701	0.934	11.797	0.642	0.904	17.013	0.256	0.626	21.559	-0.208	0.305
	(10.542,11.03)	(0.687,0.714)	(0.933,0.934)	(11.505,12.089)	(0.624,0.66)	(0.902,0.907)	(16.673,17.352)	(0.226,0.285)	(0.603,0.648)	(20.307,22.811)	(-0.34,-0.075)	(0.245,0.365)

There are mean values and 95% confidence intervals of ten replicates. The best performance is shown in bold.
